# Effects of Intraoperative Dexmedetomidine on Postoperative Pain in Highly Nicotine-Dependent Patients After Thoracic Surgery: A Prospective, Randomized, Controlled Trial

**DOI:** 10.1097/MD.0000000000003814

**Published:** 2016-06-03

**Authors:** Xingzhi Cai, Ping Zhang, Sufen Lu, Zongwang Zhang, Ailan Yu, Donghua Liu, Shanshan Wu

**Affiliations:** From the Department of Anesthesiology (XC, PZ, SL, ZZ, AY, DL SW), Liaocheng People's Hospital, Liaocheng City, Shandong; and Xuzhou Medical College (SW), Xuzhou, Jiangsu, China.

## Abstract

To investigate the effects of intraoperative dexmedetomidine on pain in highly nicotine-dependent patients after thoracic surgery.

Highly nicotine-dependent men underwent thoracic surgery and received postoperative patient-controlled intravenous analgesia with sufentanil. In dexmedetomidine group (experimental group, n = 46), dexmedetomidine was given at a loading dose of 1 μg/kg for 10 minutes, followed by continuous infusion at 0.5 μg/kg/h until 30 minutes before the end of surgery. The saline group (control group, n = 48) received the same volume of saline. General anesthesia was administered via a combination of inhalation and intravenous anesthetics. If necessary, patients were administered a loading dose of sufentanil by an anesthesiologist immediately after surgery (0 hours). Patient-controlled analgesia was started when the patient's resting numerical rating scale (NRS) score was less than 4. Resting and coughing NRS scores and sufentanil dosage were recorded 0, 1, 4 hours, and every 4 hours until 48 hours after surgery. Dosages of other rescue analgesics were converted to the sufentanil dosage. Surgical data, adverse effects, and degree of satisfaction were obtained.

Cumulative sufentanil dosage, resting NRS, and coughing NRS in the first 24 hours after surgery and heart rate were lower in the experimental compared with the control group (*P* <0.05). No patient experienced sedation or respiratory depression. Frequency of nausea and vomiting and degree of satisfaction were similar in both groups.

Intraoperative dexmedetomidine was associated with reduced resting and coughing NRS scores and a sufentanil-sparing effect during the first 24 hours after thoracic surgery.

## INTRODUCTION

The goals of postoperative pain management are to relieve pain and reduce the complications of the respiratory and circulatory systems after surgery. In this context, smoking is a predictive factor that, when present, increases the extent of postoperative pain^1^ and occurrence of perioperative complications.^2^ Smokers are required to quit smoking just before and during acute postsurgical recovery. Long-term nicotine exposure can alter the structure and function of the pain pathway.^3,4^ Furthermore, cross-tolerance between opioids and nicotine can occur in smokers.^5^ Many studies have found that abstaining smokers require larger doses of morphine^6,7^ and experience higher degrees of postoperative pain compared with nonsmokers.^7^ Similarly, we previously reported^8^ that pain severity and sufentanil requirements after surgery increased with increasing nicotine dependence. However, little research has been reported on how to improve analgesia after thoracic surgery for nicotine-dependent patients, especially highly nicotine-dependent patients.

Dexmedetomidine, a new highly selective α_2_-adrenergic receptor agonist, has sympatholytic, analgesic, and sedative properties, while lacking the side effect of respiratory depression.^9^ At the supraspinal and spinal levels, dexmedetomidine acts on the α_2_ adrenoceptors, participating in pain control through modulating nociceptive neurotransmission, inhibiting nociceptive neurons and the release of substance P. Intraoperative dexmedetomidine was shown to improve the effects of postoperative analgesia.^9,10^ However, whether the same effect is found in highly nicotine-dependent patients has not been determined. Therefore, the aim of this study was to investigate the effects of intraoperative dexmedetomidine on pain management in highly nicotine-dependent patients after thoracic surgery.

## METHODS

### Participants

Liaocheng People's Hospital Ethics Committee approval was obtained for this prospective, double-blinded, randomized controlled study, and all participants gave informed consent. The study was registered at chictr.org (ChiCTR-OCH-13004678). All patients who underwent thoracic surgery and received patient-controlled intravenous analgesia (PCIA) between May 2014 and May 2015 at Liaocheng People's Hospital were eligible for inclusion. Inclusion criteria were classification as American Society of Anesthesiologists I or II, age between 18 and 65 years, male sex, highly nicotine-dependent smoker, and ability to use PCIA and the pain number rating scale (NRS, 0 = no pain, 10 = worst possible pain). A patient was classified as a smoker if they had a >1-year history of smoking cigarettes, with a preoperative period of smoking cessation of less than 30 days. Patients who were on the nicotine patch or using nicotine nasal spray were not included. No smoking was permitted during PCIA postoperation. The Fagerstrom Test of Nicotine Dependence (FTND) was used to evaluate the extent of nicotine dependence among smokers,^11^ with an FTND score ≥6 indicating high nicotine dependence. Exclusion criteria were a body mass index greater than 30 kg/m^2^, history of chronic pain and long-term analgesic use, inability to use the PCIA device or use for less than 48 hours, renal or hepatic dysfunction, severe cardiovascular, pulmonary, or psychiatric disease, heart rate (HR) less than 45 beats per minute (bpm), type I or II atrioventricular block, and allergy to sufentanil or dexmedetomidine. The test drug was stopped and the patient was excluded from the study if the PCIA device malfunctioned, or if the patient underwent secondary surgery, went to the intensive care unit after surgery, or experienced circulatory anomaly after dexmedetomidine use without improvement after conventional treatment.

### Randomization and Blinding

An independent anesthesiologist used a computer-generated randomization table to allocate patients to treatment with saline (control group) or dexmedetomidine (experimental group). A nurse in the postanesthesia care unit (PACU) who was not one of the observers for the study prepared injectable solutions containing dexmedetomidine or 0.9% saline. Dexmedetomidine was supplied in 2-mL ampoules (200 μg), which were diluted with 48 mL of saline to a final concentration of 4 μg/mL. For patients in the control group, a 50-mL volume of 0.9% saline solution was prepared.

### Anesthesia

Before anesthesia, patients in the experimental group were administered a loading dose of 0.25 mL/kg of 1 μg/kg dexmedetomidine for 10 minutes, followed by continuous infusion at 0.125 mL/kg/h (0.5 μg/kg/h dexmedetomidine) until 30 minutes before the end of surgery. Patients in the control group received the same volume of saline. After anesthesia was induced with intravenous propofol (2 mg/kg), sufentanil (0.3 μg/kg), and cisatracurium (0.2 mg/kg), bronchial intubation was performed. Sevoflurane was inhaled to maintain anesthesia with a minimal alveolar concentration of 1.0 to 1.3 and bispectral index of 40 to 60. Cisatracurium (0.03 g/kg) was given every 1 hour from induction until approximately 1 hour before the end of the operation. Sufentanil (5 μg) was given every 30 minutes from induction until 30 minutes before the end of surgery. Intravenous ketorolac (30 mg) was given 30 minutes before the end of surgery. End-tidal carbon dioxide was maintained at 35 to 40 mm Hg. During surgery, intravenous urapidil (5–10 mg) or ephedrine (6 mg) was given if the mean arterial pressure changed beyond 20% of the base value. Atropine (0.2 mg) was administered if the HR fell below 50 bpm.

### Postoperative Analgesia Management

After surgery, patients were transferred to the PACU. If necessary, patients were administered the loading dose of sufentanil by an anesthesiologist. PCIA was started through the GemStar pump (Hospira, Lake Forest, IL) when the resting NRS was less than 4. PCIA was maintained until at least 48 hours after surgery. The PCIA device was programmed to deliver a bolus dose of 2 mL of sufentanil (0.8 μg/mL), with a lockout of 5 minutes, no background infusion, and a 4-hour limit of 30 to 40 mL of sufentanil. The goal of PCIA was to maintain the resting NRS score at less than 4. If the NRS score exceeded 4, the patient was given an additional loading dose of 3 μg of sufentanil or the bolus dose was increased from 3 to 4 mL. The 4-hour limit of 40 mL was increased to 50 mL as needed. If the NRS score at rest exceeded 6, then supplemental rescue boluses of intravenous ketorolac (30 mg) or tramadol (100 mg) were used. Patients answered a satisfaction survey after the analgesia service (Question: Are you satisfied with the postoperative analgesic services? Answer: Yes or No).

### Outcome Measures

Demographic characteristics were obtained for all patients. Pain assessment included NRS scores at rest and with coughing at arrival to the PACU (0 hours), 1 hour, 4 hours, and every 4 hours thereafter until 48 hours after surgery. Sufentanil dosage was recorded at all time points except 0 hours after surgery. Dosages of other rescue analgesics were converted to the dosage of sufentanil, based on previously described equianalgesic dose ratios (30 mg of ketorolac = 4 μg of sufentanil, 100 mg of tramadol = 10 μg of sufentanil).^12,13^ Surgical data, surgery time, anesthesia time, tracheal extubation time, and dosage of sufentanil during surgery were recorded. Hemodynamic variables, including HR, systolic blood pressure (SBP), and diastolic blood pressure, were observed during surgery. Analgesia-associated adverse effects, such as nausea, vomiting, excessive sedation, and respiratory depression, and degree of satisfaction were obtained.

### Statistical Analysis

Sample size was calculated by using PASS 11.0 (NCSS Statistical Software, Kaysville, UT). Data from our previous work gave a mean ± standard deviation (SD) for the cumulative dose of sufentanil of 120 ± 40 μg during the first 24 hours after surgery. Using a power of 80% and 2-sided significance with an α level of 0.05, a sample size of 44 patients was required. Considering a dropout rate of 15%, the final sample size was determined to be 50 patients in each group.

Data were analyzed by using SPSS 17.0 software. Descriptive statistics were used to compare clinical characteristics. Normality of data distributions was assessed with the Kolmogorov–Smirnov test. Levene test was used to determine the homogeneity of variance. Quantitative data are expressed as the mean ± SD for normal distributions or as the median [interquartile range (IQR)] otherwise. Comparisons between groups were made by using the independent-samples *t* test and Mann–Whitney *U* test, respectively. Categorical data were described as the number (%) of cases, with the *χ*^2^ or Fisher Exact Probability test being used to test differences between groups. Pain score data are shown in graphs as the mean and 95% confidence interval, with the repeated measurement method being used to compare differences between groups. A *P* value less than 0.05 was considered to be statistically significant.

## RESULTS

**FIGURE 1:**
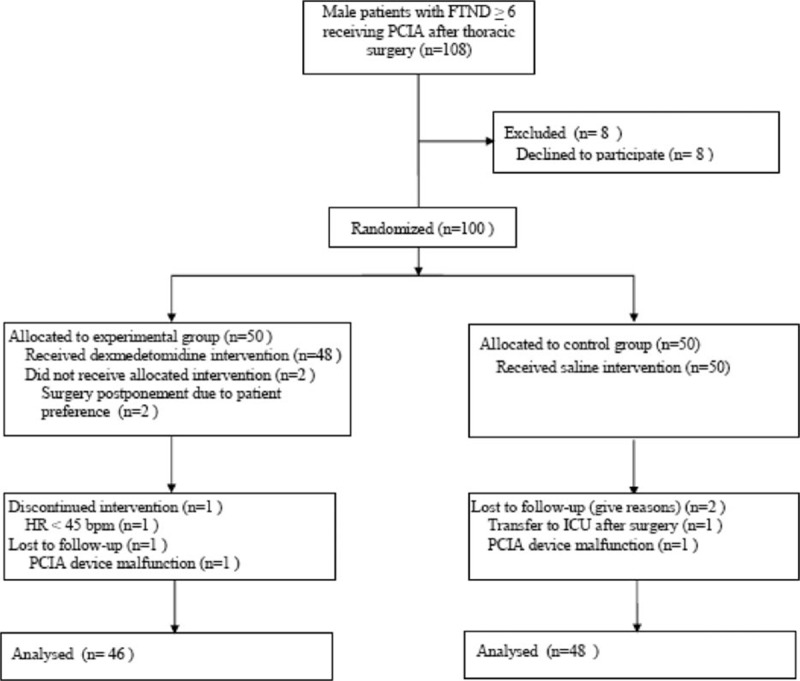
Flow chart of study selection process. FTND = Fagerstrom Test of Nicotine Dependence, ICU = intensive care unit, PCIA = patient-controlled intravenous analgesia; Experimental group, dexmedetomidine group; Control group, saline group.

The flow chart for the patient selection process is shown in Figure 1. Of the 108 recruited patients, 8 patients refused to participate in the study. Therefore, 100 patients were randomly allocated to 2 groups. In experimental group, 4 patients did not complete the study because of PCIA device malfunction (1 patient), HR less than 45 bpm (1 patient), or surgery postponement due to patient preference (2 patients). In the control group, 2 patients were excluded because of intensive care unit transfer (1 patient) or PCIA device malfunction (1 patient). Data from the remaining 94 patients were analyzed in this study.

**FIGURE 2:**
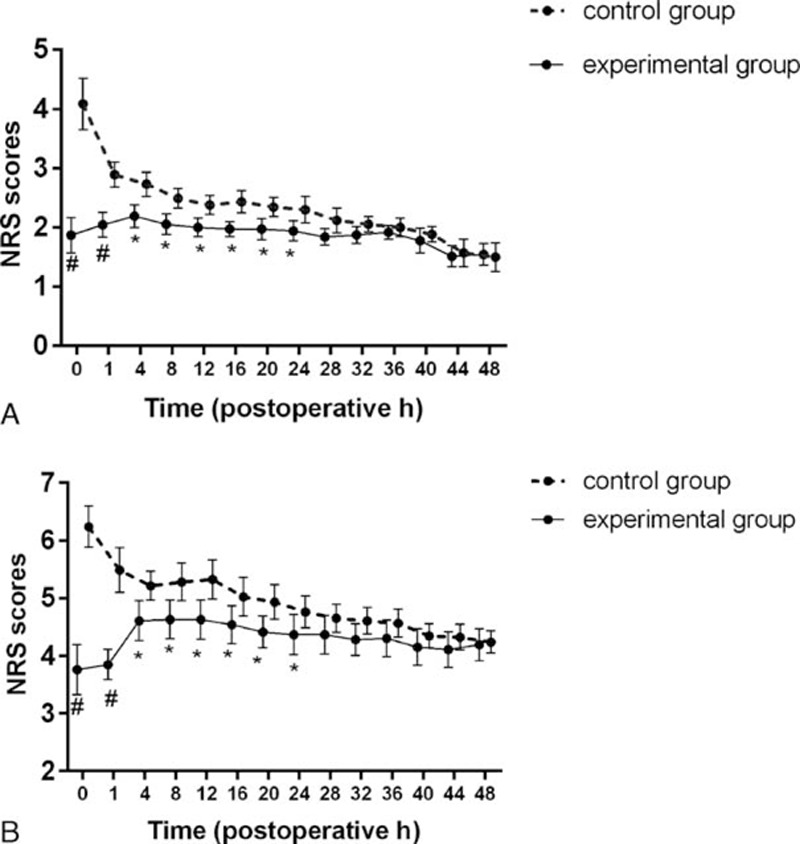
Postoperative numerical rating scale (NRS) scores at rest (A) and with coughing (B). Experimental group: dexmedetomidine group; Control group: saline group. ^∗^*P* <0.05, ^#^*P* <0.01 vs control group. Data are described using the mean and 95% confidence interval.

**FIGURE 3:**
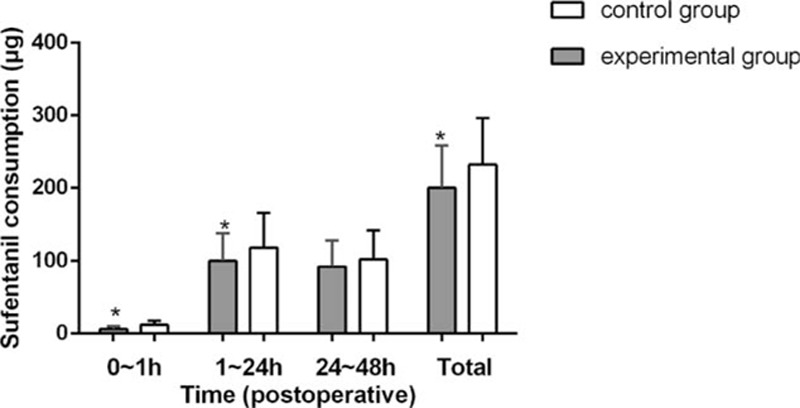
Total dosage of sufentanil throughout the period of postoperative analgesia. Experimental group: dexmedetomidine group; Control group: saline group. ^∗^*P* <0.05 vs control group.

There were no significant differences in baseline characteristics or demographics of patients, such as age, body weight, body mass index, American Society of Anesthesiologists grade, or FTND score (Table 1). Operative time, anesthesia time, awake time, and intraoperative sufentanil dosage did not differ significantly between the groups (Table 2). During the first 24 postoperative hours, the cumulative dosage of sufentanil (Figure 3), NRS at rest, and NRS with coughing (Figure 2) were significantly lower in the experimental compared with the control group; however, these values were not different between the groups beyond 24 postoperative hours (Figures 2 and 3).

**TABLE 1:**
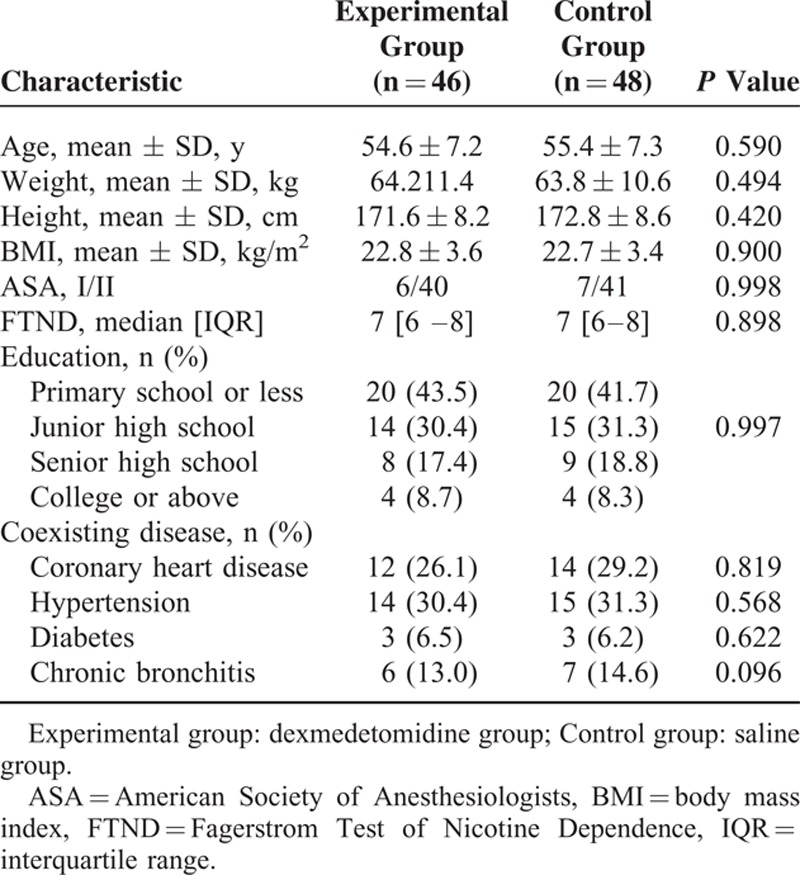
Baseline Characteristics and Demographics

**TABLE 2:**
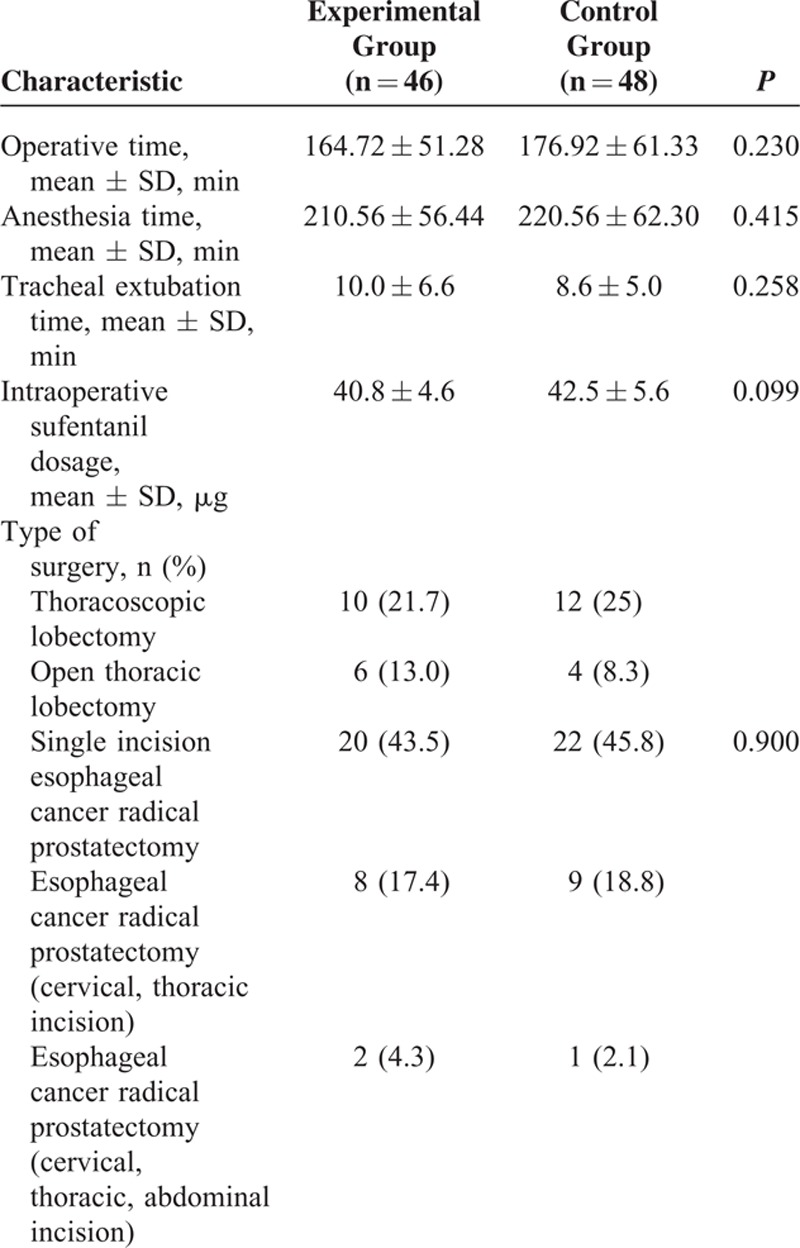
Perioperative Data

**FIGURE 4:**
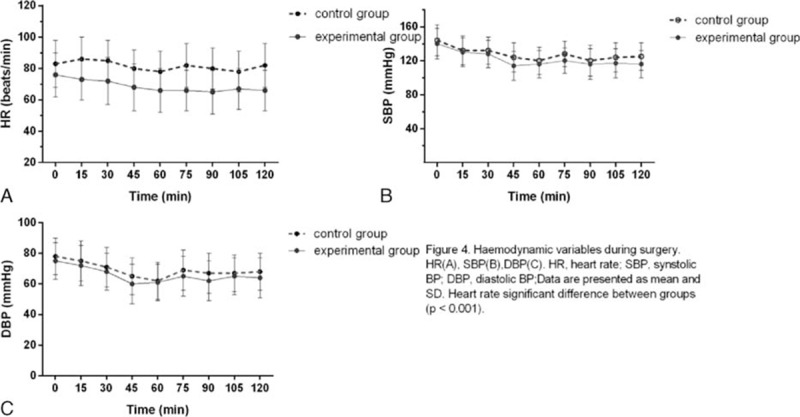
Hemodynamic variables during surgery. HR (A), SBP (B), DBP (C). DBP = diastolic blood pressure, HR = heart rate, SBP = synstolic blood pressure; data are presented as mean and SD. Heart rate significant difference between groups (*P* <0.001).

Compared with the control group, the HR of the experimental group was significantly lower, but SBP and diastolic blood pressure were not significantly different between the groups (Figure 4). No patient experienced sedation or respiratory depression. Number (%) of patients with postoperative nausea and vomiting (PONV) was 2 (4.35%) in the experimental group and 3 (6.25%) in the control group. No patient expressed dissatisfaction with the postoperative analgesia.

## DISCUSSION

This report describes the first clinical study showing that intraoperative dexmedetomidine is able to reduce the opioid requirement and pain intensity for highly nicotine-dependent subjects after thoracic surgery. The findings are consistent with the reported opioid-sparing effect of dexmedetomidine under experimental conditions.^9,14^

We evaluated the nicotine dependence of smokers according to the FTND, which includes 6 items and has a maximum possible score of 10. Smokers with an FTND score ≥6 were considered to be highly nicotine dependent.^11^ In the present study, the consumption of sufentanil was greater than in previous reports,^15,16^ due to the fact that all recruited subjects were highly dependent on nicotine. Our previous retrospective study showed that smokers with high nicotine dependence exhibited higher pain intensity and greater sufentanil usage after thoracic surgery compared with smoker patients with lower nicotine dependence.^8^ Several mechanisms may contribute to the increase in postoperative pain among smokers. Studies have shown that nicotine-dependent patients have greater pain severity (are hyperalgesic) after surgery and, thus, self-administer sufentanil more frequently to achieve analgesia.^17^ Another potential reason for the difference in sufentanil consumption is that nicotine-dependent patients might have experienced nicotine abstinence symptoms (e.g., anxiety, depressive mood), prompting greater sufentanil self-administration. Patients may have self-administered higher doses of morphine to relieve their nicotine withdrawal symptoms.

Dexmedetomidine is an α_2_-adrenergic receptor agonist that is often used for short-term sedation in patients in intensive care who are on mechanical ventilation, as an adjunct to general anesthesia, and for postoperative pain control.^18^ The main advantage of this drug is that it does not cause serious respiratory side effects. In addition, it has an opioid-sparing effect. Analgesic, sedative/hypnotic, and anxiolytic properties of dexmedetomidine make this drug potentially useful for painful surgical procedures. In some studies, intravenous dexmedetomidine during surgery was found to have a postoperative opioid-sparing effect or to reduce pain scores.^19^ These findings are consistent with our research.

Several mechanisms may contribute to dexmedetomidine-mediated reductions in pain intensity and sufentanil dosage in highly nicotine-dependent patients. First, long-term smoking induces nicotine tolerance, which facilitates tolerance to opioid receptors, thus reducing the effectiveness of opioid receptor-mediated analgesia. Dexmedetomidine was effective in postoperative pain management for opioid-tolerant patients.^20^ Second, dexmedetomidine has anxiolytic and sedative properties, and has been suggested for use in attenuating the signs and symptoms of nicotine withdrawal syndrome.^21^ As a result, dexmedetomidine use may have reduced sufentanil self-administration due to abstinence symptoms.

One research found that both dexmedetomidine and fentanyl had significant, dose-independent analgesic effects on ischemic pain, with a ceiling effect at 0.5 μg/kg.^22^ In our research, we used a dexmedetomidine dosage of 1 μg/kg for 10 minutes, followed by infusion at 0.5 μg/kg/h. This dosage provided good analgesia for at least 24 hours after thoracic surgery in patients with nicotine dependence. Many studies^9^ investigated whether dexmedetomidine can cause hypotension and bradycardia because of its dose-dependent antisympathetic effect. We did not observe any obvious hemodynamic changes among our patients. The HR dropped below 45 bpm in only 1 patient, who did not show signs of hypotension.

PONV is a common adverse side effect for patients given general anesthesia. Young age, female gender, history of PONV, high opioid dose, long anesthesia time, and certain operative types are risk factors for PONV.^23^ Being a smoker has been shown to reduce the occurrence of PONV. Although no significant difference was observed among the groups, the frequency of PONV was generally low (4.35% in the experimental group and 6.25% in the control group). This relatively low frequency of PONV may be because only male patients undergoing thoracic surgery were included in the study, and these demographics have been associated with a lower incidence of PONV. In our study, respiratory depression was not observed.

This study is limited in that some patients with nicotine dependence also had alcohol dependence, but we did not assess the latter factor. In addition, we only investigated thoracic surgery. Whether our findings are applicable to other types of surgery remains to be determined.

In summary, this prospective, randomized, controlled trial investigated the effects of intraoperative dexmedetomidine on postoperative analgesia in highly nicotine-dependent patients who underwent thoracic surgery. Intraoperative dexmedetomidine, compared with placebo, reduced NRS scores at rest and with coughing and had a sufentanil-sparing effect during the first 24 hours after thoracic surgery.
