# Effects of Intraoperative Dexmedetomidine on Postoperative Pain in Highly Nicotine-Dependent Patients After Thoracic Surgery: A Prospective, Randomized, Controlled Trial: Erratum

**DOI:** 10.1097/01.md.0000494753.90552.0a

**Published:** 2016-08-26

In the article “Effects of Intraoperative Dexmedetomidine on Postoperative Pain in Highly Nicotine-Dependent Patients After Thoracic Surgery: A Prospective, Randomized, Controlled Trial”,^1^ which appeared in Volume 95, Issue 22 of *Medicine*, two figure citations in the Results section were mislabeled. The article has since been corrected online.
